# Serum CCL1 discriminates infectious and sterile systemic inflammation in sepsis and acute pancreatitis

**DOI:** 10.1038/s41598-026-47750-w

**Published:** 2026-05-06

**Authors:** Marlies Vornhülz, Jennifer Müller, Lara Louisa Takken, Patrick Layritz, Carolin Perleberg, Jan Gärtig, Antonia Beimert, Patrick Weber, Stefan Endres, Julia Mayerle, Lesca Holdt, Ignazio Piseddu, David Anz

**Affiliations:** 1 Department of Medicine II, LMU University Hospital, Munich, Germany; 2 Institute of Clinical Pharmacology, LMU University Hospital, Munich, Germany; 3 Institute for Research in Biomedicine, Università della Svizzera italiana, Bellinzona, Switzerland; 4 Department for Orthopaedics and Trauma Surgery, LMU University Hospital, LMU Munich, Munich, Germany; 5 Institute of Laboratory Medicine, LMU University Hospital, LMU Munich, Munich, Germany

**Keywords:** Sepsis, Acute pancreatitis, Inflammation, Tregs, CCL1, CCL22, Biomarkers, Diseases, Immunology, Medical research

## Abstract

Differentiating between infectious and non-infectious etiologies in systemic inflammatory disorders may be challenging due to overlapping clinical presentations and the lack of reliable discriminating biomarkers. Regulatory T-cells (Tregs) modulate immune responses. Their functionality is governed by specific chemokines, including CCL1 and CCL22. We investigated whether these Treg-attracting chemokines are differentially regulated in infectious versus sterile inflammation. This prospective, single-center biomarker study enrolled patients with sepsis, acute pancreatitis, and hospitalized controls without infectious diseases. Serum samples were collected on days 1, 3, 5, and 7, measuring CRP, IL-6, PCT, CCL1, and CCL22. Between March 2019 and October 2022, 159 patients were enrolled, comprising 45 patients suffering from acute pancreatitis, 15 patients with confirmed sepsis as well as 99 hospitalized controls. Established inflammatory parameters CRP, IL-6 and PCT showed typical kinetics. Decreased CCL1 levels, but not CCL22, distinguished acute pancreatitis from sepsis at all time points. Additionally, CCL1 levels inversely correlated with organ failure severity in sepsis patients. CCL1 shows potential to serve as a biomarker to differentiate sterile and non-sterile inflammation in sepsis and acute pancreatitis. This may support clinical decision-making and allow a more precise use of antibiotics in these patient cohorts.

**Supplementary Information:**

The online version contains supplementary material available at 10.1038/s41598-026-47750-w.

## Background

Systemic inflammatory disorders frequently present as fulminant and potentially lethal syndromes, necessitating prompt diagnosis and immediate therapeutic intervention. Among these, both sepsis and acute pancreatitis are acute inflammatory pathologies of increasing epidemiological relevance^1,2^. Sepsis is defined as life-threatening organ dysfunction caused by a dysregulated host response to infection^3^. Organ failure is thus a crucial element of sepsis and is determined by using scoring systems such as the SOFA score^3^. Acute pancreatitis, although in most cases mild or only moderately severe^4^, may also lead to severe organ failure^5,6^. Thus, both pathologies represent critical illnesses characterized by complex immune dysregulation. Despite their overlapping clinical features, the management of sepsis and pancreatitis differs substantially. In sepsis, early administration of antibiotics is essential^3,7^, whereas antibiotics are generally not indicated in pancreatitis^5,6^. In clinical practice, differentiating between systemic inflammation due to sepsis and that due to acute pancreatitis may prove to be challenging due to their similar clinical presentation. Diagnostic differentiation is further complexified since established inflammatory serum markers, such as C-reactive protein (CRP) and interleukin-6 (IL-6), may be elevated in both pathologies, offering limited diagnostic discrimination.

Regulatory T cells (Tregs) are known to play a crucial role in the maintenance of immune homeostasis and modulation of immune responses^8–10^. Their spatial distribution and tissue recruitment during homeostasis and inflammation is orchestrated by chemokines^11^. Among these, CCL1^12,13^ and CCL22^12,14^, both of which belong to the CC chemokine family, are major Treg-attracting chemokines that govern immune regulation during homeostasis and inflammatory conditions such as infection, autoimmunity and cancer^12,13,15–21^. CCL1 signals through CCR8 and is produced mainly by activated T cells, monocytes and macrophages, promoting the recruitment of CCR8-expressing cells such as Tregs^13,22,23^. CCL22 binds to CCR4 and is predominantly secreted by dendritic cells (DCs) and macrophages, where it mediates Treg migration and facilitates Treg-DC interactions^24^. Current literature paints an ambivalent picture of Treg function in sepsis, since both favorable^25,26^ as well as unfavorable courses^27–30^ in conjunction with elevated Treg numbers have been described. Similarly, Treg function in pancreatitis appears to be conflicting, with both protective^31,32^ and negative effects^33^ reported in preclinical models. However, Tregs and their functionality cannot be quantified easily in routine practice. In contrast, their recruiting chemokines are readily detectable in serum and thus may have the potential to serve as practical diagnostic biomarkers.

To identify potential novel biomarkers which reliably discriminate between bacterial sepsis and sterile inflammation due to acute pancreatitis, we conducted a prospective, single-center, exploratory biomarker study examining Treg-attracting chemokines. We found that serum levels of CCL1 and CCL22 were independent of baseline characteristics. While CCL22 was initially suppressed in sepsis, it failed to discriminate between sepsis and acute pancreatitis. In contrast, CCL1 levels were reduced in acute pancreatitis, while they tended to be elevated in sepsis, consistently distinguishing both conditions across all measured time points. Moreover, higher CCL1 levels were inversely correlated with organ dysfunction in sepsis. Thus, CCL1 should in future be evaluated to guide clinical reasoning in patients with inflammation as well as to predict patient prognosis in sepsis.

## Results

### Study population

159 patients were enrolled between March 2019 and October 2022. Baseline characteristics of our patient cohorts are shown in Table 2. The cohort consisting of hospitalized controls comprises 99 patients. Additionally, 15 patients with confirmed sepsis and 45 patients with acute pancreatitis were included. Our patient cohort demonstrated significant differences in age, active smoking status, regular alcohol consumption as well as presence of coronary heart disease, whereas the majority of epidemiological and medical factors assessed were balanced between the groups. Among the 159 patients enrolled, serum was collected on day 1 in all cases and on day 3 in 121 cases. 24 patients with acute pancreatitis and 10 patients with sepsis had serum collected at days 1, 3 and 5. The median observation period was three days.

**Table 1:** Inclusion and exclusion criteria.

Inclusion criteria	Patients with confirmed sepsis	Patients with sterile inflammation	Patients with non-inflammatory disorders
	confirmed sepsis = confirmed infection AND increase of SOFA-Score ≥ 2 points	acute pancreatitis post-operative inflammation	non-inflammatory disorders, e.g. decompensated liver cirrhosis, cardiac insuffiency
Exclusion criteria	Malignant disease fever of unknown origin acute coronary syndrome	Malignant disease confirmed infection fever of unknown origin acute coronary syndrome	

**Table 2:** Baseline characteristics. A total of 159 patients were included in the analysis. Data are presented as n (%) or median (range). The p-values for age and BMI were calculated by one way analysis of variance (ANOVA), all the others were calculated by Chi2 test.

Characteristics	Patient groups			*p*-value	
	Controls (*n* = 99)	Sepsis (*n* = 15)	Acute Pancreatitis (*n* =45)		
Age [years]	67 (23–90)	73 (23–93)	50 (21–81)	0,0016	(*)
Sex				0,1375	
Female	34 (34%)	9 (60%)	15 (33%)		
Male	65 (66%)	6 (40%)	30 (67%)		
BMI [kg/m^2^]	26,3 (16,2–42,2)	25,7 (17,3–41,5)	26,2 (13,8–40,5)	0,4790	
Smoking status					
Current	16 (16%)	1 (7%)	13 (29%)	0,0219	(*)
Past	21 (21%)	5 (33%)	7 (16%)	0,0545	
Drinking status					
Regular alcohol consumption	19 (19%)	5 (33%)	15 (33%)	0,0479	(*)
Diabetes mellitus	17 (17%)	5 (33%)	9 (20%)	0,2911	
Arterial Hypertension	36 (36%)	7 (47%)	20 (44%)	0,4889	
Coronary Heart Disease	30 (30%)	6 (40%)	3 (7%)	0,0026	(*)
Thyroid disease	12 (12%)	3 (20%)	4 (9%)	0,4709	
Sepsis etiology	n/a		n/a		
Urosepsis		7 (47%)			
Pneumonia		2 (13%)			
Other		6 (40%)			
Pancreatitis Etiology	n/a	n/a			
Biliary			16 (36%)		
Alcohol			10 (22%)		
Hypertriglyceridaemia			3 (7%)		
Drug-induced			2 (4%)		
Idiopathic			11 (24%)		
Other			3 (7%)		

Regarding disease etiology, the most frequent source of sepsis was the urinary tract (urosepsis, *n* = 7), followed by pneumonia (*n* = 2). The remaining patients presented with mixed or other etiologies. Blood cultures were positive in 9 of 15 patients (60%). In the acute pancreatitis cohort, the most common etiologies were biliary (*n* = 16) and alcoholic (*n* = 10), with the remaining patients presenting with mixed etiologies (*n* = 8) or idiopathic (*n* = 11).

Of the 15 sepsis patients, 10 were admitted to ICU or IMC at day 1, while 2 patients were not escalated to higher-level care based on medical or personal reasons, and 3 patients were managed outside these units at the time of enrollment. The median overall SOFA score at day 1 was 11 (IQR 9–11). ICU/IMC-admitted patients had a median SOFA score of 11 (IQR 10–13), whereas patients not admitted to ICU/IMC had a median SOFA score of 8.5.

### Established inflammatory serum markers show typical kinetics in our cohort

Patients presenting with sepsis and acute pancreatitis are known to have elevated inflammatory serum markers such as CRP, PCT and IL-6^5,34,35^. We measured these parameters in our cohorts on day 1, 3, 5 and 7 (Fig. 2). Medians and interquartile ranges of these parameters are shown in Suppl. Table 1. As expected, CRP was significantly elevated compared to controls in both groups at all measured time points, with higher levels in sepsis patients compared to pancreatitis patients at disease onset (Fig. 2A)^34^. PCT showed significant elevation compared to controls at all measured time points with significantly higher values in sepsis patients on all days (Fig. 2B). IL-6 levels were elevated only on the first three days of both types of inflammation, with highest values in sepsis patients on day 1 (Fig. 2C). Taken together, established inflammatory parameters show the kinetics expected from a clinician’s perspective^34,35^, confirming the representability of our patient cohort.

**Fig. 1:**
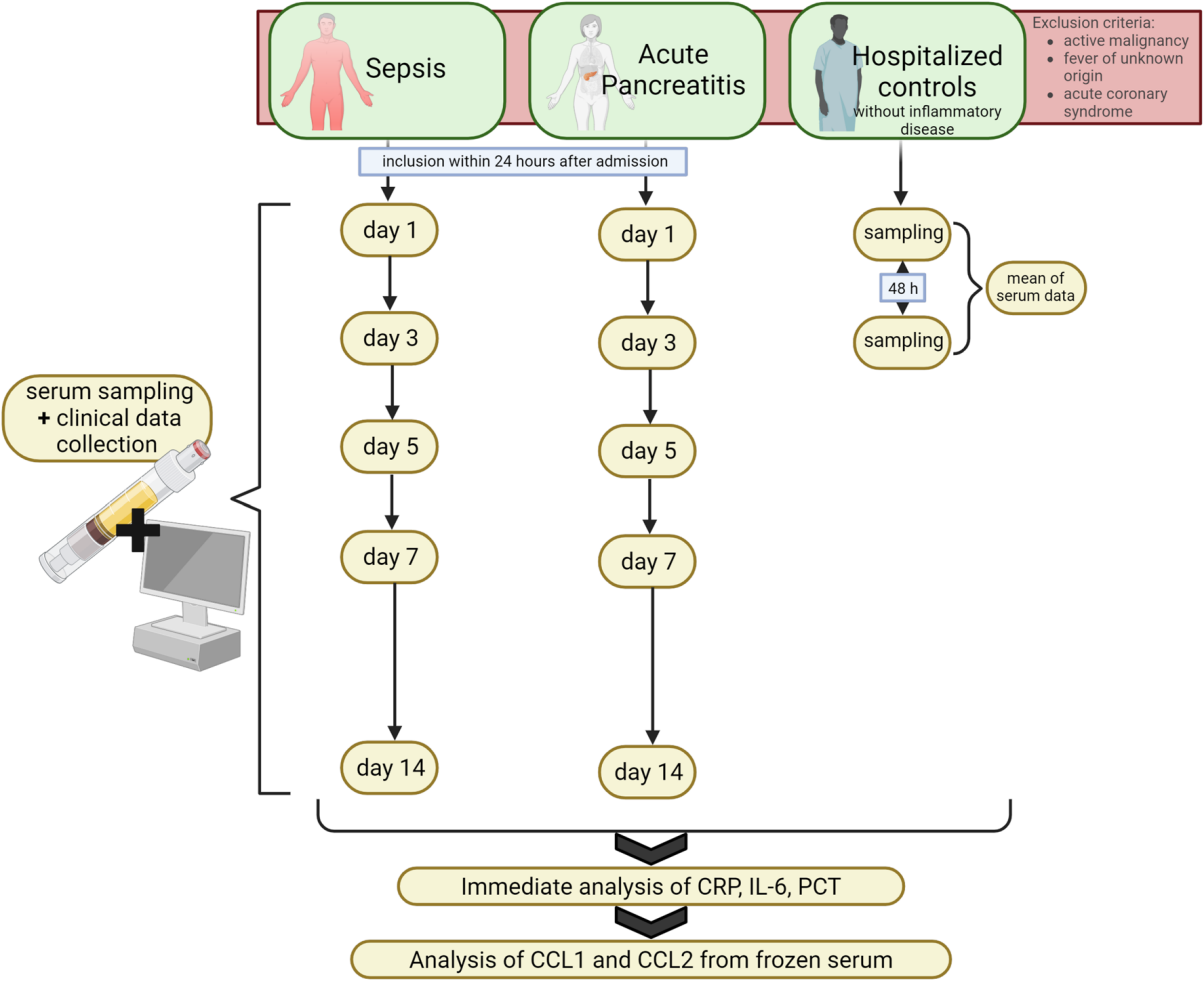
Study Design. A representative study flow chart is shown. Figure created using BioRender.com

**Fig. 2:**
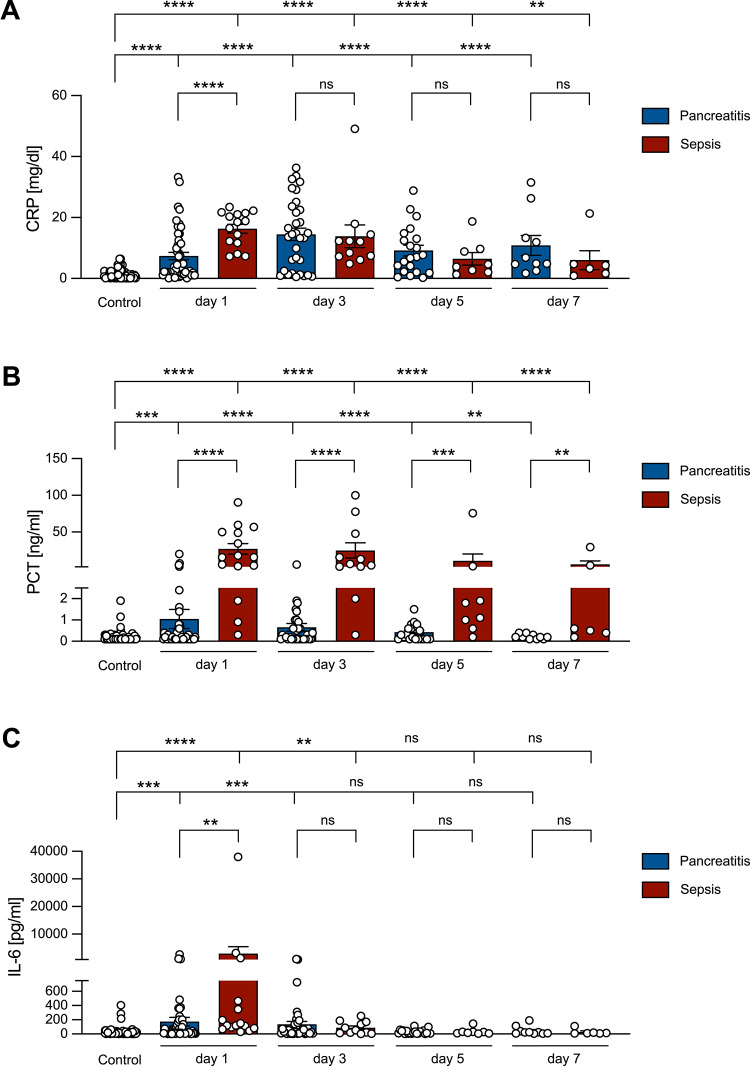
Established inflammatory serum markers show typical kinetics in our cohort. Serum levels of (**A**) CRP, (**B**) PCT and (**C**) IL-6 of hospitalized controls (control) and patients with pancreatitis and sepsis were determined at the indicated time points after hospital admission. Graphs show mean ± SEM. Statistical analysis was performed using two-tailed Mann-Whitney-U test

### CCL1 potently discriminates acute pancreatitis and sepsis

**Fig. 3:**
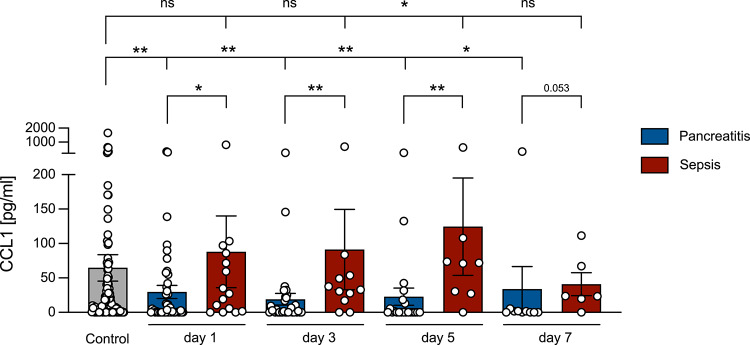
CCL1 potently discriminates acute pancreatitis and sepsis. CCL1 serum levels of hospitalized controls (control) and patients with pancreatitis and sepsis were determined at the indicated time points after hospital admission. Graphs show mean ± SEM. Statistical analysis was performed using two-tailed Mann-Whitney-U test

To determine whether Treg-attracting chemokines can serve as diagnostic tools to distinguish between pancreatitis and sepsis, we compared the levels of the chemokines CCL1 and CCL22 between our patient cohorts. All medians and interquartile ranges of these parameters are displayed in Suppl. Table 1. CCL1 was significantly suppressed in acute pancreatitis at all measured time points (Fig. 3). In sepsis, there appears to be a tendency towards elevated CCL1 levels on the first five days, although statistically significant only on day 5. Consequently, acute pancreatitis and sepsis were significantly discriminated by CCL1 levels in the first five days (Fig. 3). CCL22 levels, on the other hand, remain unaffected by inflammation in acute pancreatitis (Suppl. Figure 1). CCL22 levels were significantly suppressed in comparison to controls on days 1 and 3 of sepsis (Suppl. Figure 1), as published previously by our group^36^ and others^37^. However, CCL22 was not able to differentiate between acute pancreatitis and sepsis. To further explore the potential diagnostic performance of CCL1, we performed exploratory receiver operating characteristic (ROC) analyses comparing sepsis and acute pancreatitis at the respective time points. CCL1 demonstrated moderate to good discriminative performance, with the highest area under the curve (AUC) observed at day 5 (AUC 0.812), while earlier time points showed lower AUC values (Suppl. Figure 2). However, given the exploratory nature of this study and the limited size of the sepsis cohort, these analyses should be interpreted cautiously.

Since some baseline characteristics, such as age, smoking status and the presence of coronary heart disease, demonstrated significant differences between our cohorts (see Table 2), we tested all these characteristics for differences in the distribution of serological values. Smokers presented significantly higher PCT values and trended towards increased IL-6 levels (Suppl. Figure 3), while CRP levels were significantly elevated in diabetics (Suppl. Figure 4). In contrast, none of these baseline characteristics was associated with significant differences in serum CCL1 or CCL22 levels (Suppl. Figures 3 and 4). Furthermore, considering the different etiologies of acute pancreatitis and sepsis, we could not identify any correlation leading to significantly different CCL1 or CCL22 levels on the first day of disease in our cohorts (Suppl. Figure 5). Taken together, serum CCL1 levels potently discriminate between acute pancreatitis and sepsis. Additionally, important demographic and medical baseline characteristics did not influence serum CCL1 levels, indicating its applicability in clinical settings.

### High CCL1 is associated with less organ failure in sepsis patients

**Fig. 4:**
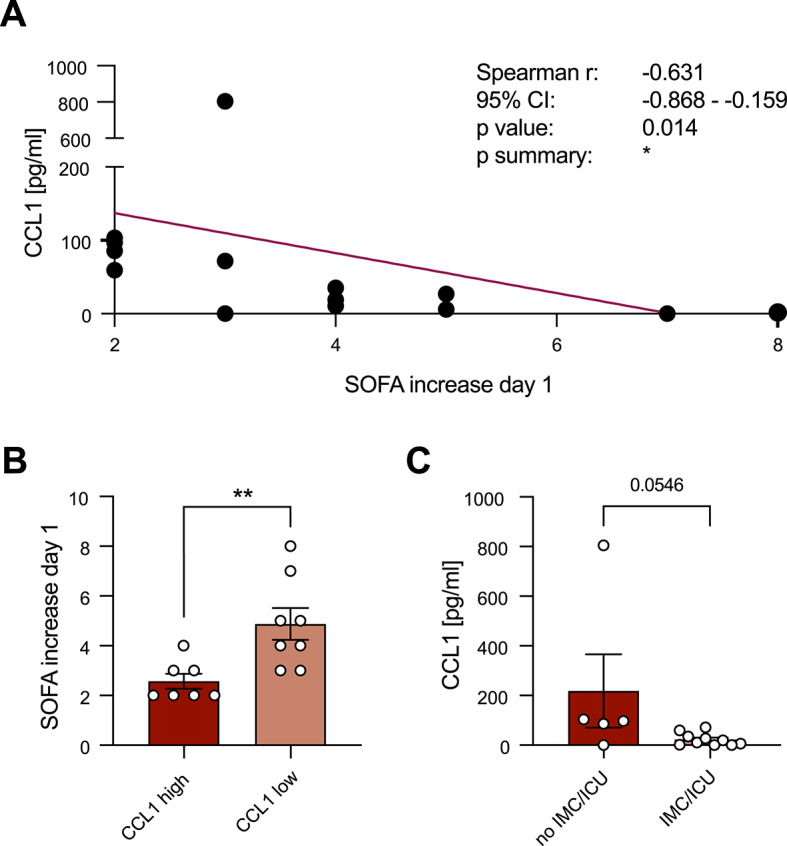
High CCL1 is associated with less organ failure in sepsis patients. (**A**) CCL1 serum levels and SOFA score increase of sepsis patients at day 1 were determined and plotted against each other. Afterwards, Spearman correlation was performed. (**B**) Sepsis patients were subdivided into CCL1high and CCL1low patients, according to the median. SOFA increase on day 1 was then determined for both groups. Graph shows mean ± SEM. Statistical analysis was performed using two-tailed Mann-Whitney-U test. (**C**) CCL1 serum levels in sepsis patients admitted to intermediate care (IMC) or intensive care unis (ICU) and patients treated on normal wards (no IMC/ICU) were determined. Graph shows mean ± SEM. Statistical analysis was performed using two-tailed Mann-Whitney-U test

We were further interested whether serum CCL1 is associated with organ failure in sepsis patients. Indeed, CCL1 levels inversely correlate to organ dysfunction, as measured by SOFA score increase at onset in sepsis patients (Fig. 4). Since only one sepsis patient died within the observation period, predictions of the mortality in relation to chemokine levels were not possible. However, we determined the correlation between serum CCL1 and patients’ SOFA score increase on the first day of sepsis as a proxy for the severity of organ failure. Here, serum CCL1 levels inversely correlated with SOFA score increase, meaning that patients with higher CCL1 levels had lower SOFA score increases at disease onset (Spearman r: -0.63, 96% CI -0-87 - -0.16, *p* = 0.014) (Fig. 4A). Accordingly, the increase of SOFA scores on the first day of sepsis was significantly higher in patients with low CCL1 levels compared to patients with high CCL1 levels (Fig. 4B). In line with this, sepsis patients who were not admitted to an intensive care unit (ICU) or intermediate care unit (IMC) trended towards higher serum CCL1 values than those admitted to ICU or IMC (Fig. 4C). Altogether, serum CCL1 levels correlate with sepsis severity, suggesting a so far unknown biological role of CCL1 in sepsis immune response.

## Discussion

In this prospective biomarker study, we evaluated established inflammatory markers alongside the Treg-attracting chemokines CCL1 and CCL22 in a cohort of 159 patients with sepsis, acute pancreatitis and hospitalized controls. Notably, CCL1 levels showed differing trends between acute pancreatitis and sepsis across all measured timepoints, with discriminative ability progressively improving and reaching its highest level on day 5. Moreover, higher CCL1 levels showed a possible association with reduced early organ dysfunction in sepsis, suggesting a potential prognostic role that requires validation in larger cohorts. Combined with its discriminative ability between sepsis and acute pancreatitis, CCL1 emerges as a candidate biomarker of translational interest.

To our knowledge, systemic CCL1 levels have not previously been studied in patients with sepsis or acute pancreatitis. Previous data have linked elevated CCL1 levels to autoimmune pancreatitis^38^ and other autoimmune diseases^13,20,23,39–41^, whereas high expression of CCR8, representing the receptor for CCL1, on peripheral blood mononuclear cells (PBMCs), was shown to be associated with severe acute pancreatitis of various etiologies^42^. The mechanisms underlying the differential regulation of serum CCL1 levels in acute pancreatitis and sepsis in our study remain unclear. Acute pancreatitis is primarily driven by sterile inflammation induced by DAMPs released from injured acinar cells, whereas sepsis is initiated by infection and involves strong PAMP-driven innate immune activation^43,44^. These distinct upstream signals may generate different chemokine programs. In this context, alveolar macrophages were shown to upregulate CCL1 via Wnt/β-catenin signaling in a murine sepsis model^45^, a pathway unlikely to be activated in sterile inflammation. Consistent with this, CCL1 was also identified among the chemokines induced by Group B Streptococcus membrane vesicles in a human macrophage cell line upon NLRP3 inflammasome activation^46^.

Moreover, CCL1 signals through CCR8, which is expressed on regulatory T cells, and CCL1-CCR8 interactions can promote Treg recruitment and suppressive function^13^. We therefore speculate that reduced circulating CCL1 in acute pancreatitis may reflect reduced systemic induction and/or increased tissue-level consumption of CCL1, whereas higher CCL1 levels in sepsis may reflect stronger immune activation in response to infection-associated stimuli and may be linked to regulatory pathways that modulate excessive inflammation and organ dysfunction. These interpretations remain hypothetical, as this study did not include cellular immune profiling such as PBMC analysis or direct assessment of CCR8 expression and Treg distribution. However, our study is the first to report systemic CCL1 levels in both conditions and to contextualize these findings within an emerging mechanistic framework.

This study reports patients with sepsis and acute pancreatitis along with a large group of hospitalized controls. In the past, controls have often been collected from healthy donors, which might not adequately reflect the inflammatory serum levels in hospitalized patients. Nevertheless, serum CCL1 levels showed notable heterogeneity within the hospitalized control cohort, which may influence the interpretation of circulating chemokine levels and suggests that clinically applicable cut-off values would require validation in larger cohorts. Despite this variability, the inclusion of hospitalized controls provides a more clinically relevant comparison than healthy donors.

Due to its high clinical need, diagnostic parameters to differentiate sepsis from non-infectious inflammation have already been extensively studied in the past. Numerous biomarkers have been evaluated, however the established parameters CRP and PCT often turned out to have the best discriminating potential despite their limitations^35^. Interpretation of PCT can yet be challenging in certain clinical settings, including advanced kidney disease, major surgery, trauma or other non-infectious inflammatory conditions, where PCT levels may be elevated independently of bacterial infection^47^. In such situations, additional biomarkers reflecting different aspects of the host immune response may provide complementary diagnostic information. Treg-associated chemokines have not been previously explored in this context. Consistent with the observed differences in circulating CCL1 levels between sepsis and acute pancreatitis, exploratory receiver operating characteristic (ROC) analyses demonstrated moderate to good diagnostic performance of CCL1 for distinguishing both conditions, with the highest AUC observed at day 5. While future diagnostics may rely on multimodal approaches or AI-supported algorithms, evaluation of specific single parameters is essential for healthcare at present. This stresses the translational relevance of CCL1 as discriminating factor in patients with acute pancreatitis and sepsis. In the context of acute pancreatitis, a relevant application could be in biliary pancreatitis, where early identification of concurrent cholangitis is crucial to guide antibiotic use and ERCP^5,48^. The relatively small number of patients in our sepsis cohort reflects the strict inclusion criteria of this prospective study and limited recruitment during routine clinical practice. Consequently, the present study was not powered for formal diagnostic validation or determination of clinically applicable diagnostic cut-off values, and the ROC analyses should therefore be interpreted as exploratory. Thus, future studies should investigate the utility of CCL1 in this setting.

Tregs, in contrast to their attracting chemokines, have been studied in preclinical pancreatitis models, while findings remain conflicting. A mouse model of acute pancreatitis revealed that depletion of CD4^+^ T cells surprisingly suppressed disease severity^33^, an effect attributed to a reduced intrapancreatic Th2 response rather than changes in Treg numbers. In contrast, more recent preclinical data support a protective role of Tregs: Tregs were shown to potently suppress the proinflammatory response in a preclinical model of acute pancreatitis^49^, and Galectin-3 deficiency was found to attenuate pancreatic injury by promoting intrapancreatic Foxp3 + Treg accumulation and IL-10 production^32^. Corroborating these experimental findings, a Mendelian randomization study identified resting CD45RA+ Tregs as partial mediators of a genetically determined protective effect against acute pancreatitis^31^. However, clinical data remain conflicting: whereas elevated Treg numbers in peripheral blood have been associated with less organ failure^50^, another study reported increased Treg counts specifically in patients with poor outcome^51^. Pharmacological approaches aimed at enhancing Treg activity, such as Ulinastatin^52^, which elevates Treg numbers and thereby ameliorates the course of acute pancreatitis, have shown benefit not only in preclinical settings^53^ but also clinically^52^. These divergent findings suggest a complex, context-dependent role of Tregs in pancreatitis pathophysiology, both suppressing intrapancreatic and systemic inflammation, but ultimately resulting in possible detrimental immunosuppressive bystander effects. Therefore, the pathogenetic role of the CCL1-CCR8 axis and its impact on local and systemic Treg functionality in acute pancreatitis requires further investigation in preclinical pancreatitis models.

Strikingly, we observed elevated CCL1 levels to be associated with reduced organ dysfunction in sepsis. One possible explanation is that high CCL1 levels reflect effective immune activation due to CCL1 induction upon immune cell activation, supporting bacterial clearance and preventing secondary organ injury. Alternatively, higher CCL1 concentrations may enhance CCR8-dependent recruitment and activation of Treg, thus modulating excessive inflammation and potentially limiting organ injury. This dual interpretation aligns with current literature, where Tregs have been associated with both favorable and unfavorable outcomes in sepsis^25–29^. It yet remains a compelling observation that CCL1 appears to be relevant for organ function in sepsis, while its blood levels remain unaltered. Functional studies in murine sepsis models as well as human patients regarding Treg distribution and activity are required to answer the mechanistic background of this phenomenon observed in our cohort.

In summary, CCL1 may facilitate clinical reasoning in patients with inflammation and thus contribute to a more precise use of antibiotics in this patient collective. The inverse correlation between CCL1 levels and organ dysfunction in sepsis further suggests a prognostic role, consistent with data indicating a protective function of Tregs during sepsis. Future research yet remains to elucidate the function of CCL1 in systemic inflammation caused by acute pancreatitis and sepsis. An additional promising avenue would be to explore CCL1 as a discriminating biomarker in transplant patients, where differentiation between allograft rejection and infectious complications remains diagnostically challenging. Beyond diagnosis, Tregs and their attracting chemokines may represent druggable targets to attenuate harmful hyperinflammatory responses.

## Methods

### Study design

We designed a prospective, monocentric, diagnostic study to evaluate Treg-associated chemokines and other laboratory and clinical biomarkers as well as follow-up parameters over the course of seven days after hospital admission. The study design is illustrated schematically in Fig. 1, which was created using BioRender (BioRender.com) and can be accessed using the following link: https://BioRender.com/kb6a9vp.

Three cohorts of patients were included in the study (Table 1): patients with confirmed diagnosis of sepsis, patients with sterile inflammation due to acute pancreatitis and hospitalized control patients with non-inflammatory diseases. Patients with active malignancy, fever of unknown origin and acute coronary syndrome were excluded. Additionally, confirmed infection led to exclusion in case of sterile inflammation as well as controls. Sepsis was defined as confirmed infection (such as pneumonia or cystitis) as well as an increase of SOFA score of at least 2 points, according to the Sepsis-3 consensus guidelines^3^. Patients in the sepsis and sterile inflammation groups were included within 24 h after hospital admission. Patients’ written informed consent was obtained in all cases.

### Blood collection

The first blood collection was performed on the first morning after hospital admission. Blood was collected on day 1, day 3, day 5, day 7 and day 14, unlike the patient was discharged before. Blood from hospitalized controls was collected at two to three timepoints with 48 h interim period in each case.

### Measurement of laboratory values and chemokines

Patients’ serum was transferred to the LMU Institute of Laboratory Medicine. C-reactive protein (CRP), interleukin-6 (IL-6) and procalcitonin (PCT) were measured according to routine practice of the institute. Another part of the patients’ serum was aliquoted and frozen at − 80 °C. Subsequently, CCL1 and CCL22 serum levels were determined by ELISA according to the manufacturer’s protocols (Human CCL1/I-309 DuoSet ELISA #DY272, Human CCL22/MDC DuoSet ELISA #DY336, both R&D Systems). For patients in the control cohort, all serologic parameters were measured on at least two separate time points with an interval of at least 48 h, and mean values of all measured time points were created to obtain control values for serologic markers.

### Documentation of clinical data

Along with the blood collection, clinical data of patients was documented pseudonymized on days 1, 3, 5, 7, where applicable, in an electronic case report form (eCRF) provided by the Institute of Laboratory Medicine.

### Statistics

Statistical analysis was performed using Microsoft Office Excel (Version 16.75.2) as well as R (Version 4.3.1), R Studio (Version 2023.06.0 + 421) and GraphPad Prism Version 10. The following R packages were used: tidyverse, readxl, dplyr, lubridate, writexl, ggplot2, ggpubr, ggsignif, nnet, and pROC. Significance was determined by Wilcoxon test, unless otherwise specified. The measured values of controls were tested for significant inter-individual differences using a two-tailed Student’s *t* test and since not significant, individual means were calculated and taken as control values. Statistical significance was defined as follows: * = *p* < 0.05, ** = *p* < 0.01, *** = *p* < 0.001 and **** = *p* < 0.0001. Receiver operating characteristic (ROC) curve analysis was performed to assess the discriminatory performance of serum CCL1 between sepsis and acute pancreatitis at days 1, 3 and 5. Sepsis was defined as the positive outcome and acute pancreatitis as the negative outcome. Area under the curve (AUC) values were calculated for each timepoint.

### Ethics

The study was reviewed and approved by the LMU ethics committee on December 12th, 2018 (internal record 698 − 16). All participants provided informed consent prior to participation in the study.

## Supplementary Information

Below is the link to the electronic supplementary material.

Supplementary Material 1

## Data Availability

The datasets used and/or analyzed during the current study are available from the corresponding author on reasonable request. Data will be provided in anonymized form to protect participant confidentiality.

## Abbreviations

CCL1: Chemokine (CC motif) ligand 1
CCL22: Chemokine (CC motif) ligand 22
CRP C: Reactive protein
ELISA: Enzyme linked immunosorbent assay
ICU: Intensive care unit
IMC: Intermediate care unit
IL-6: Interleukin-6
MDC: Macrophage-derived chemokine, alternate name for CCL22
PCT: Procalcitonin
SOFA: Sepsis-related organ failure assessment
Treg: Regulatory T-cell

## Declarations

### Data availabilitiy

The datasets used and/or analyzed during the current study are available from the corresponding author on reasonable request. Data will be provided in anonymized form to protect participant confidentiality.
