# Correction: Serum CCL1 discriminates infectious and sterile systemic inflammation in sepsis and acute pancreatitis

**DOI:** 10.1038/s41598-026-58739-w

**Published:** 2026-06-22

**Authors:** Marlies Vornhülz, Jennifer Müller, Lara Louisa Takken, Patrick Layritz, Carolin Perleberg, Jan Gärtig, Antonia Beimert, Patrick Weber, Stefan Endres, Julia Mayerle, Lesca Holdt, Ignazio Piseddu, David Anz

**Affiliations:** 1 Department of Medicine II, LMU University Hospital, Munich, Germany; 2 Institute of Clinical Pharmacology, LMU University Hospital, Munich, Germany; 3 Institute for Research in Biomedicine, Università della Svizzera italiana, Bellinzona, Switzerland; 4 Department for Orthopaedics and Trauma Surgery, LMU University Hospital, LMU Munich, Munich, Germany; 5 Institute of Laboratory Medicine, LMU University Hospital, LMU Munich, Munich, Germany

Correction to: *Scientific Reports* 10.1038/s41598-026-47750-w, published online 06 May 2026

The original version of this Article contained an error in the Figures where blurry images were published.

The original Figures 1, 2, 3, and 4 and accompanying legends appear below.

**Fig. 1:**
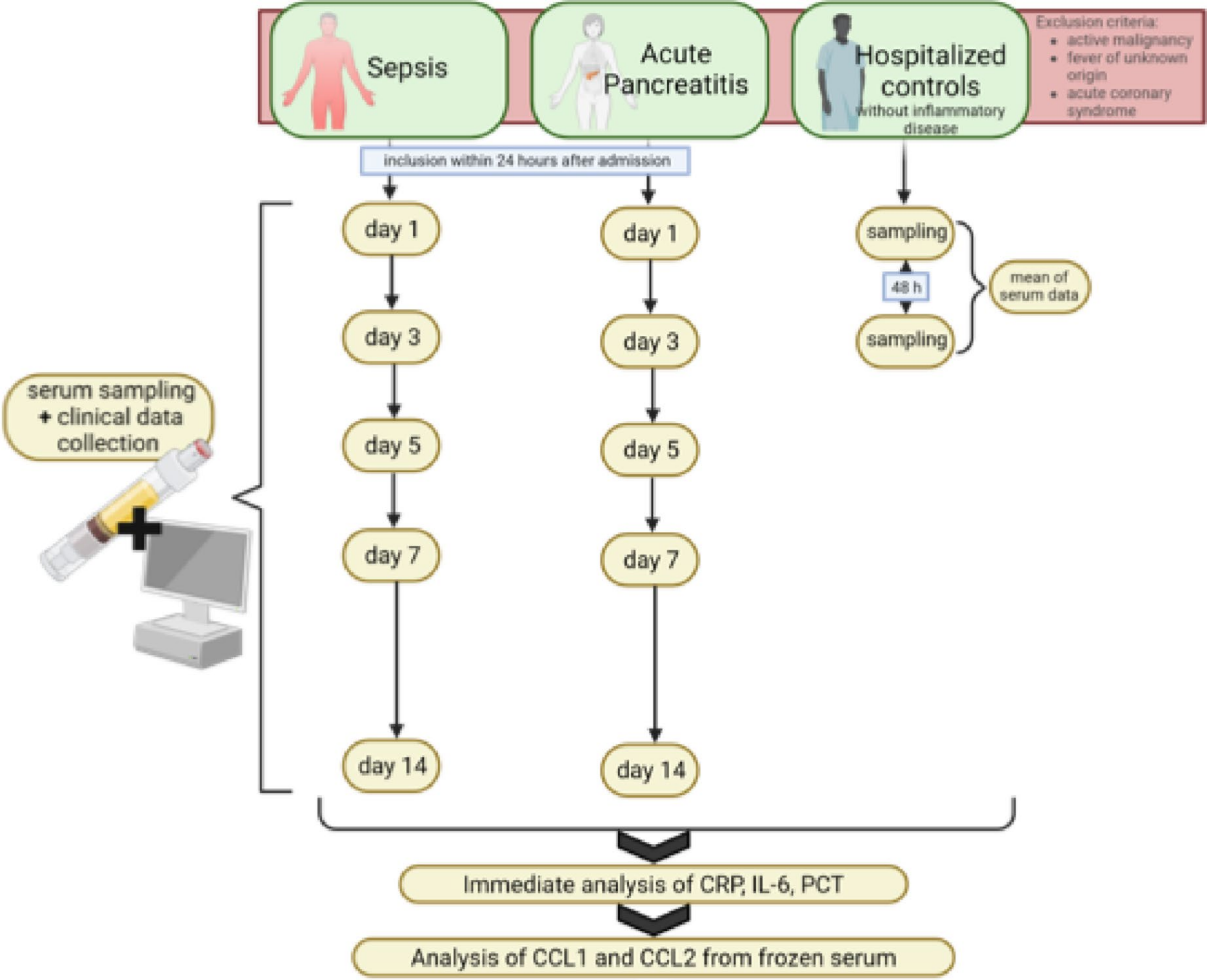
Study Design. A representative study flow chart is shown. Figure created using BioRender.com.

**Fig. 2:**
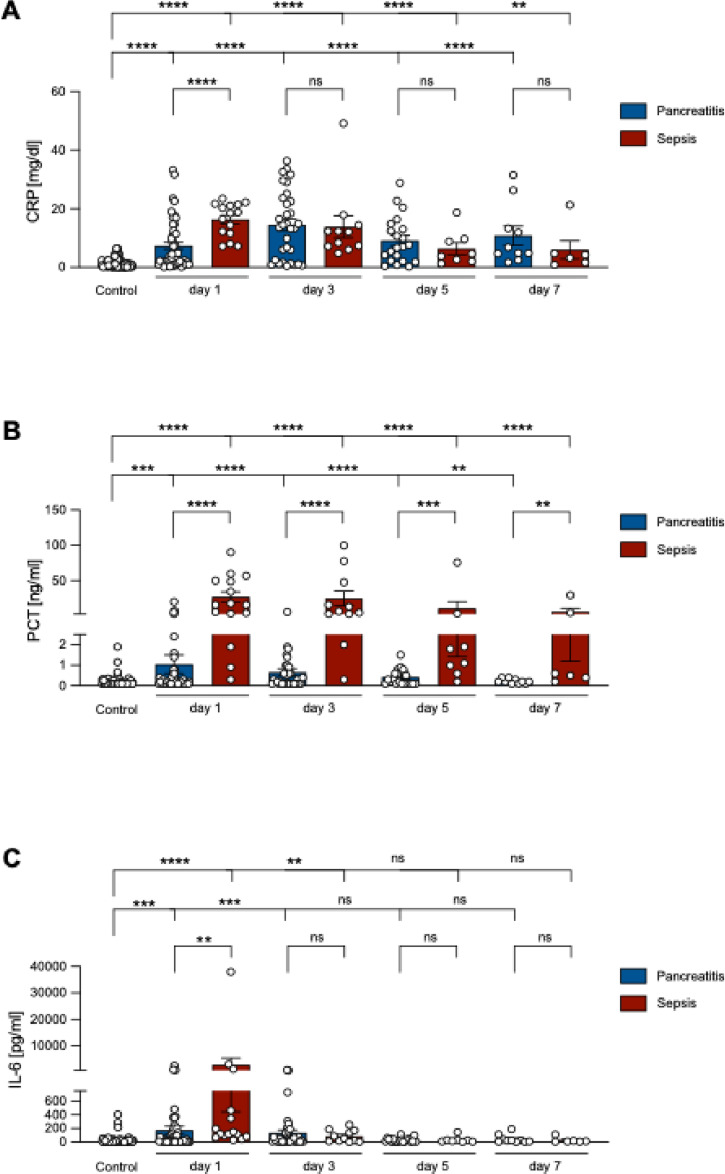
Established inflammatory serum markers show typical kinetics in our cohort. Serum levels of (A) CRP, (B) PCT and (C) IL-6 of hospitalized controls (control) and patients with pancreatitis and sepsis were determined at the indicated time points after hospital admission. Graphs show mean ± SEM. Statistical analysis was performed using two-tailed Mann–Whitney-U test.

**Fig. 3:**
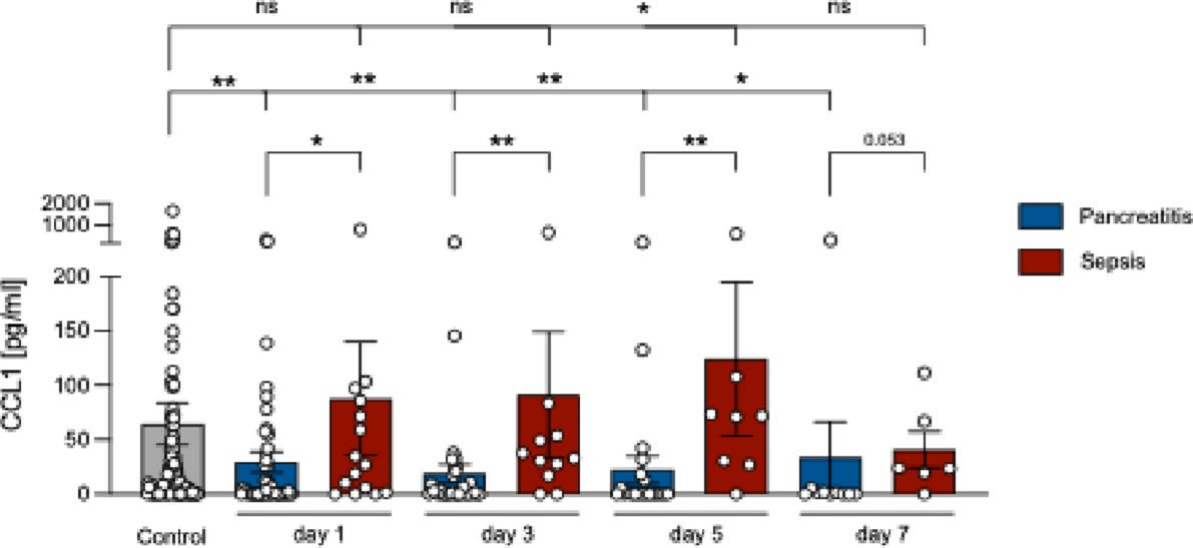
CCL1 potently discriminates acute pancreatitis and sepsis. CCL1 serum levels of hospitalized controls (control) and patients with pancreatitis and sepsis were determined at the indicated time points after hospital admission. Graphs show mean ± SEM. Statistical analysis was performed using two-tailed Mann–Whitney-U test.

**Fig. 4:**
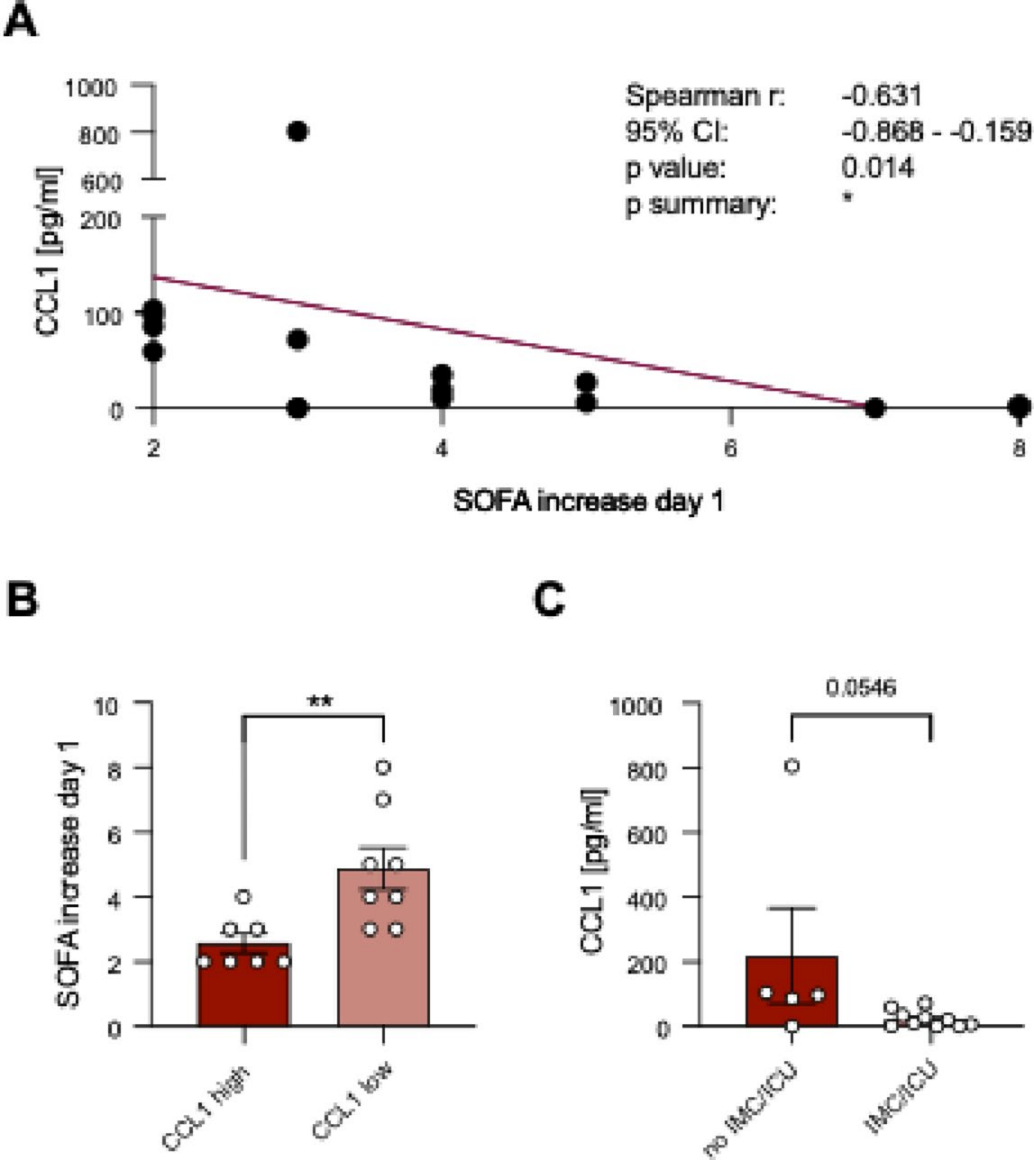
High CCL1 is associated with less organ failure in sepsis patients. (**A**) CCL1 serum levels and SOFA score increase of sepsis patients at day 1 were determined and plotted against each other. Afterwards, Spearman correlation was performed. (**B**) Sepsis patients were subdivided into CCL1high and CCL1low patients, according to the median. SOFA increase on day 1 was then determined for both groups. Graph shows mean ± SEM. Statistical analysis was performed using two-tailed Mann–Whitney-U test. (**C**) CCL1 serum levels in sepsis patients admitted to intermediate care (IMC) or intensive care unis (ICU) and patients treated on normal wards (no IMC/ICU) were determined. Graph shows mean ± SEM. Statistical analysis was performed using two-tailed Mann–Whitney-U test.

The original Article has been corrected.

