# Supplementary material for: Serum CCL1 discriminates infectious and sterile systemic inflammation in sepsis and acute pancreatitis
Source: Sci Rep. 2026 May 6;16:14391. doi: 10.1038/s41598-026-47750-w (PMC13149532; doi:10.1038/s41598-026-47750-w)
Supplement: Supplementary file 1 — Supplementary Material 1 [file 41598_2026_47750_MOESM1_ESM.pdf]

Supplementary Material

Supplementary Table 1: Serum parameters in patient groups.

Values are presented as median (interquartile range).

Parameter	unit	Control	Acute Pancreatitis				Sepsis			
			Day 1	Day 3	Day 5	Day 7	Day 1	Day 3	Day 5	Day 7
**CRP**	mg/dl	0,75 (0,23 - 1,58)	3,8 (1,3 - 11,4)	14,05 (2,3 - 22,78)	7,0 (2,2 - 13,0)	5,85 (4,73 - 12,95)	18,5 (11,9 - 21,2)	12,1 (7,3 - 13,4)	4,2 (2,53 - 9,03)	3,75 (2,0 - 4,6)
**Procalcitonin**	ng/ml	0,1 (0,1 - 0,12)	0,2 (0,1 - 0,6)	0,25 (0,1 - 0,68)	0,3 (0,1 - 0,7)	0,2 (0,13 - 0,28)	15,7 (3,9 - 45,5)	6,4 (2,95 - 31,7)	1,45 (0,9 - 2,28)	0,55 (0,43 - 3,6)
**Interleukin-6**	pg/ml	10,75 (4,6 - 25,23)	22,6 (9 - 169)	30,4 (8,9 - 144)	17,2 (7,8 - 42,5)	22,9 (7,45 - 35,38)	120 (87,4 - 406,5)	53,7 (15,8 - 153)	20,8 (10,2 - 29,7)	12,7 (7,7 - 16,6)
**CCL1**	pg/ml	11,23 (1,22 - 45,33)	1,69 (0 - 30,79)	1,69 (0 - 17,87)	0 (0 - 12,8)	0,84 (0 - 1,83)	27,11 (3,66 - 78,69)	32,98 (22,42 - 51,45)	71,49 (29,71 - 82,28)	23,6 (20,6 - 56,3)
**CCL22**	pg/ml	217,8 (174,1 - 319,6)	235,4 (148,9 - 324,8)	204,9 (156,1 - 284,3)	204,0 (153,6 - 322,7)	196,2 (124,2 - 257,2)	175,2 (137,7 - 226,6)	163,2 (122,8 - 226,2)	214,5 (162,2 - 300,5)	252,4 (188,8 - 276,4)

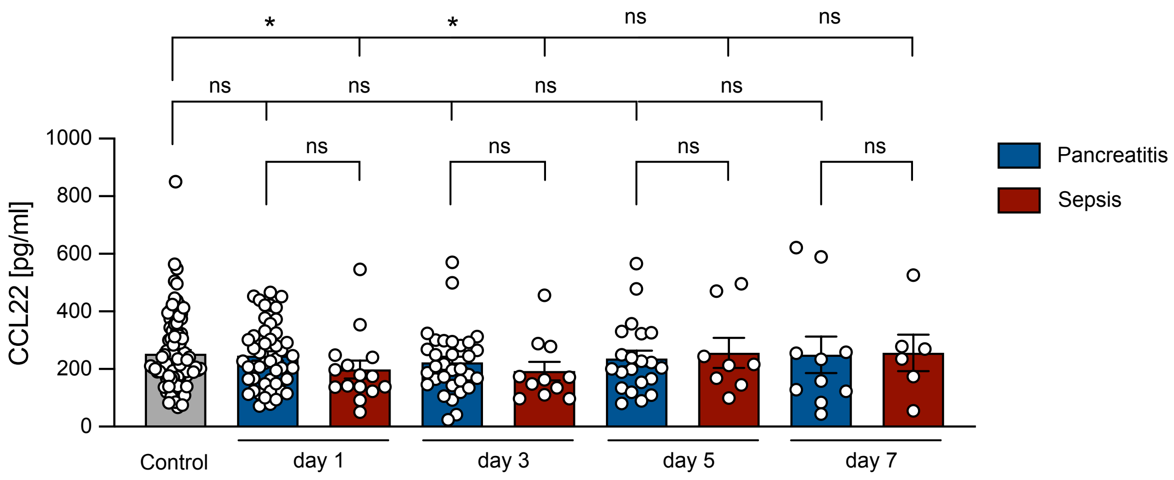

Supplementary Figure 1: CCL22 does not discriminate pancreatitis and sepsis.

CCL22 serum levels of hospitalized controls (control) and patients with pancreatitis and sepsis were determined at the indicated time points after hospital admission. Graphs show mean ± SEM. Statistical analysis was performed using two-tailed Mann-Whitney-U test.

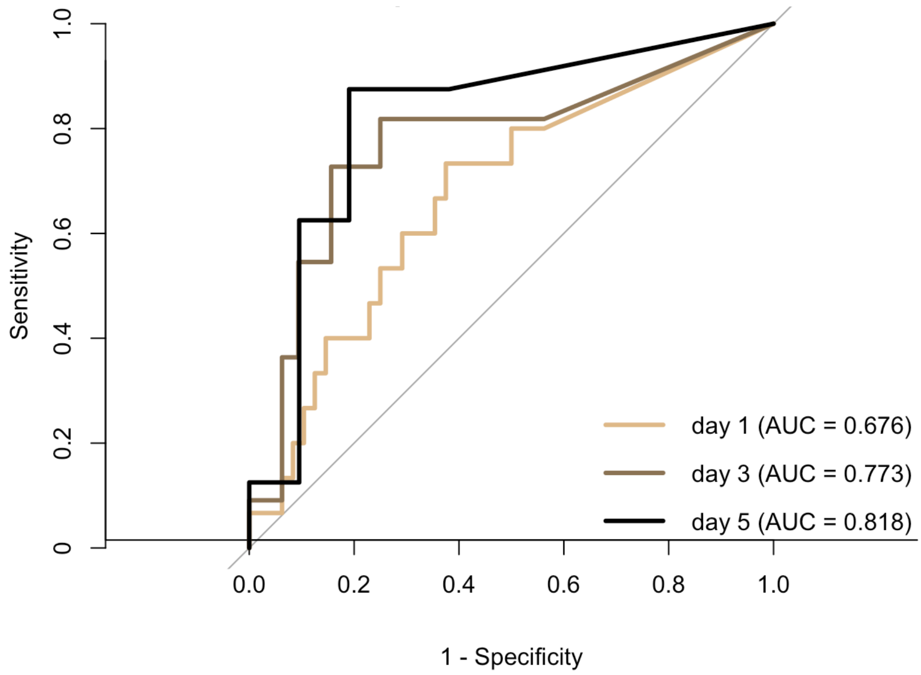

Supplementary Figure 2: ROC curve analysis of serum CCL1 for discrimination of sepsis and acute pancreatitis.

Receiver operating characteristic (ROC) curves for serum CCL1 levels discriminating sepsis from acute pancreatitis at day 1 (light blue, AUC = 0.676), day 3 (blue, AUC = 0.773) and day 5 (dark blue, AUC = 0.818). The diagonal grey line represents the line of no discrimination.

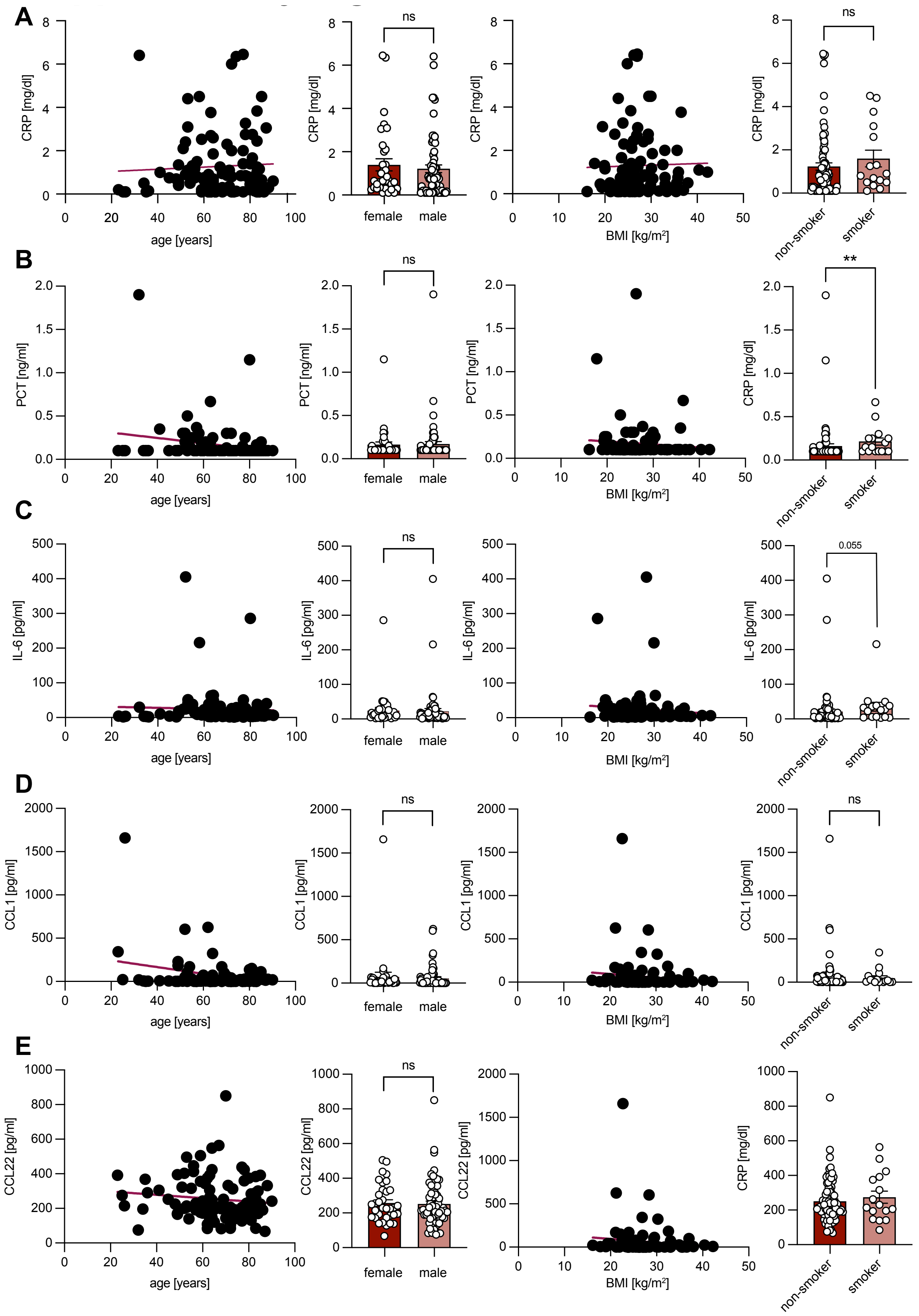

Supplementary Figure 3: Serum markers are not affected by epidemiological characteristics of individuals.

Serum levels of (A) CRP, (B) PCT, (C) IL-6, (D) CCL1 and (E) CCL22 of hospitalized controls were determined by ELISA and correlated with age, gender, BMI and smoking status. Age and BMI correlations show individual values. Gender and smoking status associations are displayed as mean ± SEM and statistical analysis was performed using two-tailed Mann-Whitney-U test.

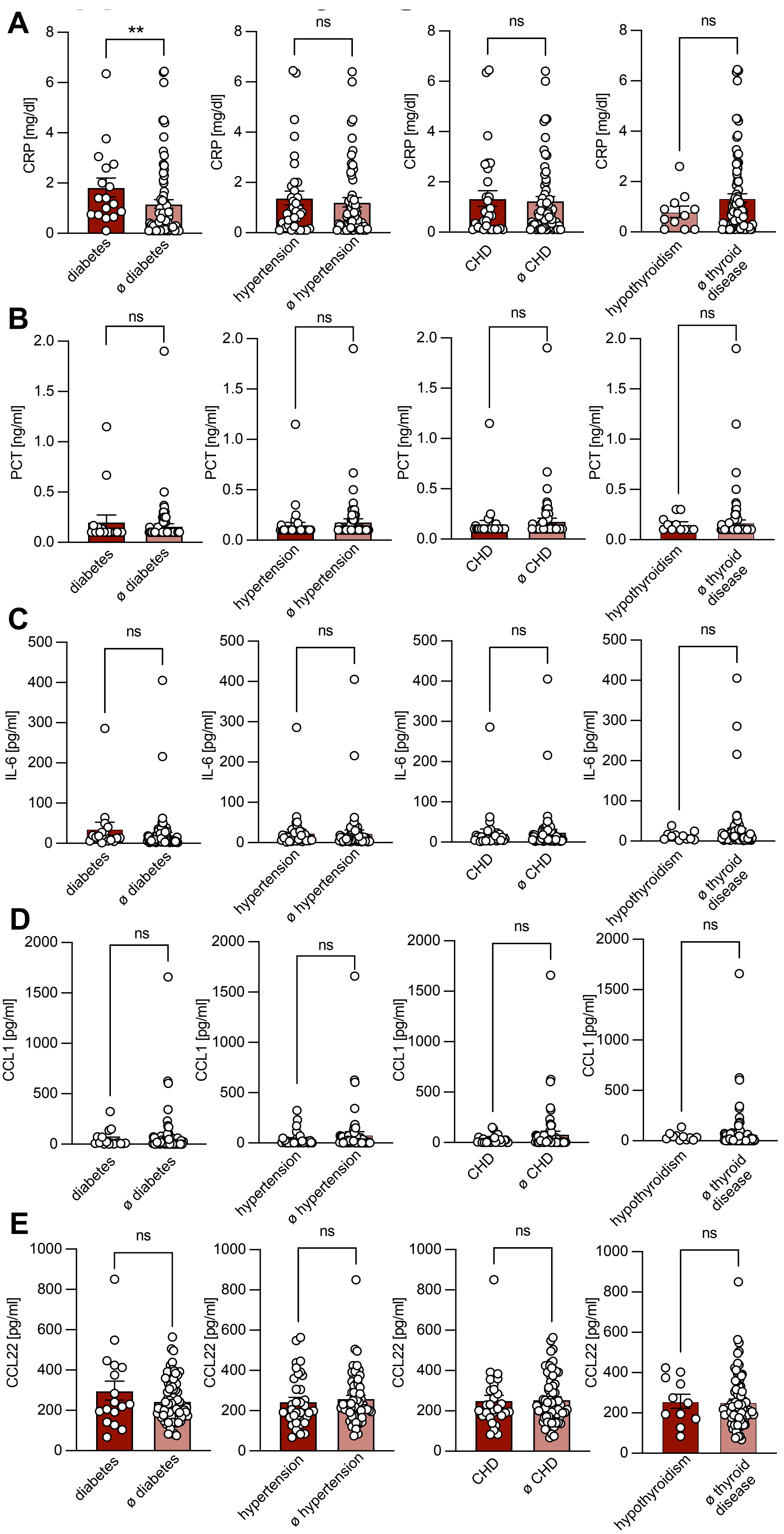
A
B
C
D
E
CRP [mg/dl]
PCT [ng/ml]
IL-6 [pg/ml]
CCL1 [pg/ml]
CCL22 [pg/ml]
**
ns
diabetes
ø diabetes
hypertension
ø hypertension
CHD
ø CHD
hypothyroidism
ø thyroid disease

Supplementary Figure 4: Serum markers are not affected by medical history of individuals. Serum levels of (A) CRP, (B) PCT, (C) IL-6, (D) CCL1 and (E) CCL22 of hospitalized controls were determined by ELISA and correlated with diabetes, hypertension, coronary heart disease (CHD) and thyroid disease, as indicated. Graphs show mean ± SEM. Statistical analysis was performed using two-tailed Mann-Whitney-U test.

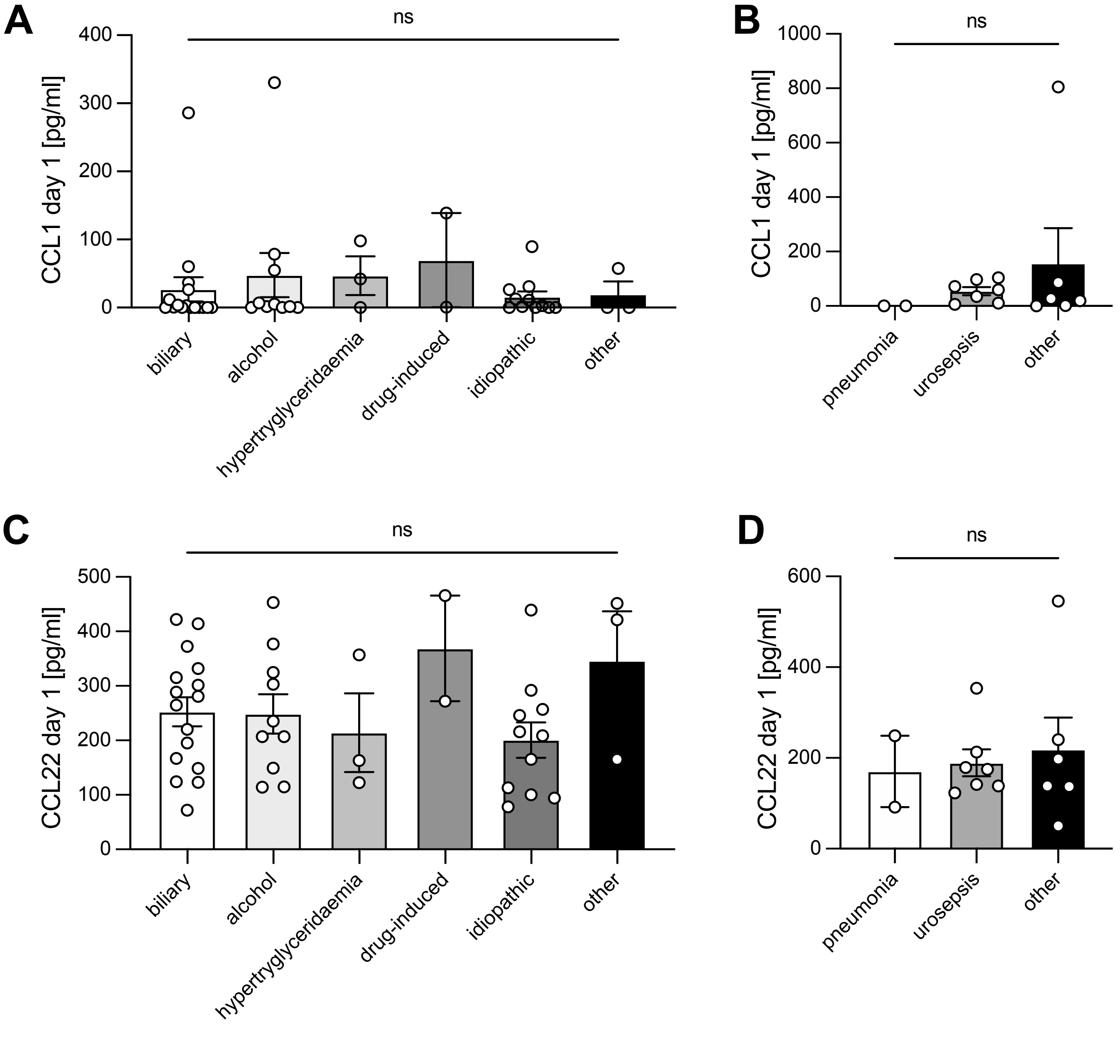

Supplementary Figure 5: Chemokine levels are not affected by etiology of pancreatitis or sepsis. Serum levels of CCL1 (A and B) and CCL22 (C and D) on day 1 after hospital admission were determined by ELISA in pancreatitis and sepsis patients, respectively. Patients were then subdivided according to etiology of pancreatitis (A and C) and sepsis (B and D), respectively. Graphs show mean ± SEM. Statistical analysis was performed using Kruskal-Wallis test with Dunn's multiple comparisons test.
